# Involving Families with Osteogenesis Imperfecta in Health Service Research: Joint Development of the OI/ECE Questionnaire

**DOI:** 10.1371/journal.pone.0147654

**Published:** 2016-01-22

**Authors:** Maman Joyce Dogba, Noémi Dahan-Oliel, Laurie Snider, Francis H. Glorieux, Michaela Durigova, Telma Palomo, Michel Cordey, Marie-Hélène Bédard, Christophe Bedos, Frank Rauch

**Affiliations:** 1 Department of Family and Emergency Medicine, Faculty of Medicine, Université Laval, Québec City, Quebec, Canada; 2 Office of Clinical Research, Shriners Hospital for Children, Montreal, Quebec, Canada; 3 School of Physical and Occupational Therapy, McGill University, Montreal, Quebec, Canada; 4 Genetics Unit, Shriners Hospital for Children, Montreal, Quebec, Canada; 5 Faculty of Dentistry, McGill University, Montreal, Quebec, Canada; 6 Department of Social and Preventive Medicine, Faculty of Medicine, Université de Montréal, Montreal, Quebec, Canada; IRCCS E. Medea, ITALY

## Abstract

**Background:**

Despite the growing interest in understanding the psycho-social impact of rare genetic diseases, few studies examine this concept and even fewer seek to obtain feedback from families who have lived the experience. The aim of this project was to involve families of children living with osteogenesis imperfecta (OI) in the development of a tool to assess the impact of OI on the lives of patients and their families.

**Methods:**

This project used an integrated knowledge translation approach in which knowledge users (clinicians and people living with OI and their families) were consulted throughout the four steps of development, that is: content mapping, item generation, tool appraisal and pre-testing of the questionnaires. The International Classification of Functioning and Health was used as a framework for content mapping. Based on a scoping review we selected two validated tools to use as a basis for developing the questionnaire. The final parent self-report version measured six domains: experience of diagnosis; use of health services; use of social and psychological support services; expectations about tertiary specialized centers; and socio-demographic information.

**Results:**

A total of 27 out of 40 families receiving care at the Shriners Hospital for Children-Canada and invited to participate in the pre-test returned the completed questionnaires. In more than two-thirds of families (69%; *n* = 18) OI was suspected either at or within the first 3 months after birth. Up to 46% of families consulted between 3 and 5 doctors (46%; n = 12) prior to final diagnosis. The use of services by families varied from 0 to 16 consultations, 0 to 9 exploratory examinations and 1 to 10 types of allied health services. In the 12 months prior to the study, fewer than a quarter of children had been admitted, for treatment, for hospital stays of longer than 8 hours or to an emergency department (24% and 9% respectively). Only 29% of parents received psychological support.

**Conclusion:**

This joint development process generated a tool, with good psychometric properties, that provides unique insight into the experiences of patients and families with OI, the psycho-social impact of the illness, and their service needs and expectations.

## Introduction

Osteogenesis Imperfecta (OI) is a rare genetic disorder characterized by increased bone fragility that affects approximately 1 in 10,000 people [1, 2]. Clinical signs include both skeletal (including fractures that can occur with trivial or no trauma, short stature, limb deformities) and extra-skeletal symptoms (e.g., teeth abnormalities, hearing disorders). Seven clinically defined types of OI are currently recognized (OI types I to VII), but other genetically defined types have also been reported [2]. The diagnosis and treatment of severe forms of OI often require complex care provided by multidisciplinary teams, and are therefore the focus of this paper [3].

As with other rare genetic diseases (RGDs), OI can take a severe toll on family function and organization, and can increase the cost and burden of care on the health care system [4, 5]. There is a large body of research on the biomedical aspects of OI. However, interest in the psycho-social effects of OI is recent [6, 7] in part because of the relatively small proportion of individuals affected by the disease [8–10]. Previous studies on this topic have demonstrated that parents of children with OI experience high levels of stress because of difficulties obtaining an accurate diagnosis that, in turn, result in delays accessing appropriate services [5, 11]. In addition, parents report that the pressures of caring for children with OI impact family dynamics and organization, and often lead to social isolation. Furthermore, while patients and the families of severely affected children report that they often need intense medical and social support services following diagnosis, little is known about the extent to which they are able to access these services. For example, researchers in one study found that people living with OI had difficulty accessing specialized services such as physical therapists with knowledge of the disease and respite care [12]. However, the results of this exploratory qualitative study cannot be generalized to the broader population of patients and families living with OI. There is therefore a dearth of evidence on the pre- and post-diagnosis experiences, challenges and expectations of families living with OI and their service needs.

Today health service research is characterized by an ethos of partnership where patients, caregivers and the public are expected to be full and active participants. Moreover, evidence suggests that “for patients with rare conditions, research *is* care” (http://www.rarediseasefoundation.org/) and patients and families with RGDs are eager to be involved in the research and development process because they believe they have a contribution to make [13]. Indeed, findings of previous studies on OI show that parents acquire unique knowledge of the disease through caring for their children, but often feel that their expertise is neither acknowledged nor valued by health care professionals [11, 14]. The present study is embedded within a larger project that uses an integrated knowledge translation approach to involve patients and families living with OI in assessing the impact of OI on their lives [15]. This paper reports on the systematic development of a tool to assess the experience of diagnosis, the pattern and use of services, and the challenges and service expectations of families living with OI.

## Methods

Ethical approval was obtained from the McGill Institutional Review Board (A00-B45-13A). Participants in the pre-test provided written informed consent. This study was conducted at the Shriners Hospital for Children (SHC) in Montreal, Canada, a specialized pediatric orthopedic hospital affiliated with McGill University.

The project team was composed of researchers (MJD, ND-O, LS, CB), clinician scientists (FR, JN, FG), one patient living with OI (MC) and the caregiver of a child with OI (M-HB). We used an integrated knowledge translation (IKT) approach [16] to involve knowledge users (clinicians, and people living with OI and their families) in all four stages of development of the tool, specifically: content mapping, item generation, tool appraisal and pre-test of the questionnaires. In addition to the project team, an advisory committee composed of clinicians who were also on the research team and a patient and a caregiver, was formed to bolster patient input in the development process. The patient and the caregiver were selected because of their interest in participating in this research project at the SHC. We chose these two individuals in order to have a native French speaking and a native English speaking person on the committee. As members of the advisory committee, they participated in the initial in-person meeting to set up the project. Later they revised early versions of the questionnaire and provided feedback to the research team via emails or phone calls. The patient and caregiver received a lump sum compensation of $40 in appreciation of their time. All project and advisory committee members contributed to reporting the findings of this project.

### Stage 1: Content mapping

The World Health Organization’s International Classification of Functioning and Health (ICF) [17] was the theoretical framework used to guide the development of the questionnaire. In addition to drawing on findings from our earlier research on the quality of life of patients and families with OI [11, 14], we conducted an ad hoc scoping review on the determinants of quality of life in order to identify key areas to be included in the questionnaire. Six areas were retained for the final questionnaire: experience of the diagnosis, use of health services, use of social and psychological support services, expectations regarding tertiary specialized centers, participation in research and socio-demographics.

The patient and the caregiver did not participate in this first stage because we could not provide them with training in scoping reviews due to resource constraints.

### Stage 2: Item generation

The review allowed us to identify validated tools to use in the development of a questionnaire tailored to the experiences of individuals with rare genetic disorders (such as OI) and their families. We focused on tools that were generic, self-report measures that could be answered either by patients and/or their families, and were easy to complete. We selected two tools that met these criteria and could be adapted with the authors’ permission. The first tool is a questionnaire used in a large European survey about the experiences and expectations of patients with over 40 rare diseases. [9], The second tool, the “Impact On Family scale (IOF)” [18] has been widely used to assess the impact of chronic childhood conditions on families. A recently validated, shorter, 15-item version of the original questionnaire [19] was selected for the present study. Finally, the research team added questions about the frequency of use of health, social and psychological support services, and participation in research.

### Stage 3: Validation of the questionnaire

The questionnaire was named I-OI/ECE (Impact of OI, Experiences, Challenges and Expectations of patients and families). The initial versions of the questionnaire (I-OI/ECE. 1.0) were first revised by three clinicians (a family physician, a pediatric bone specialist and a physician himself affected by OI) working in the field of OI to ensure content validity and that the items were pertinent to the OI population. The revised questionnaire (I-OI/ECE 1.1) was then submitted to the advisory committee for review. Next, the I-OI/ECE 1.2 was translated into French using a forward-backward translation process [20, 21]. We categorized health service use as hospital admissions (hospital stay > 8 hours), visits to an outpatient clinic, and visits to the emergency department [7].

### Stage 4: Questionnaire pre-test

Forty parents, each with a child diagnosed with OI who was being treated or followed at SHC, were invited to pre-test the parent version of the questionnaire. To be eligible to participate, parents had to be able to read English or French and be willing to give signed informed consent. In cases where families had more than one child affected by OI, the research team consensually agreed that the questionnaire would target the oldest child. While there were no exclusion criteria, we intentionally invited a larger proportion of families with severe OI, based on the assumption that they have a heavier burden of care. A research assistant used SHC medical records to identify participants meeting the inclusion criteria. The project leader or a research assistant called potential participants and invited them to participate in the study. During the call the project leader or research assistant described the study in detail, discussed potential risks and benefits, and answered any questions that were asked. Participants who agreed to participate received a study package containing the consent form, questionnaire, instruction sheet, and a prepaid return envelope that was either mailed or handed to them at a regular clinical visit.

Three weeks after initial contact, the research assistant made a reminder call to participants who had not yet returned the questionnaire. After the questionnaires were completed, a research assistant followed up with families to assess the time required to complete the questionnaire and evaluate the clarity of items.

### Data analysis

The IBM Statistical Package for the Social Sciences (SPSS-22) software was used to analyze the quantitative data. The internal consistency of the questionnaire was assessed using Cronbach alphas for the global IOF Scale, and the Family/Social (FS) and Personal Strain (PS) subscales. Informal discussions were held with the patient and the caregiver to gather their insights about their experience participating in the research project.

## Results

### Stages 1 to 3

The development process took place over a 6-month period (November 2013–April, 2014) resulting in four final versions of the questionnaires: a self-report version for patients (available in French and English) and a parent-report version for parents (also available in French and English). The final English parent-report version (I-OI/ECEp 2.0) was 24 pages long and contained 7 sections (experience of the diagnosis, use of healthcare services, use of social and psychological services, impact on families scale, participation in research, expectations about specialized services, socio-demographic data). Based on comments from the patient and caregiver on the advisory team, the patient self-report version contained all the above sections except for the experience of the diagnosis because in most cases of severe OI, the diagnosis was made during childhood.

### Stage 4

Of the 40 parents invited to pre-test the parent-report version of the (I-OI/ECEp 2.0), 27 returned the completed questionnaire. The majority of questionnaires (68%; *n* = 17) were completed by the mother alone. Demographics of respondents and the target children are shown in Table 1.

**Table 1:** Characteristics of respondents and their children diagnosed with OI*.

Characteristics of children diagnosed	
	N(%)
**Gender**	
Male	12(44.4)
Female	14(51.9)
**Age of child at time of study (in years)**	
0–10	10(43.5)
11–20	10(43.5)
21–30	3(13.0)
Type of OI*	
I	3(11.5)
III	3(11.5)
IV	10(38.5)
V	3(11.5)
VI	1(3.8)
VII	1(3.8)
Other	5(19.2)
**Characteristics of respondents**	
**Age of mother**	
21–30	1(4.3)
31–40	9(39.1)
41–50	9(39.1)
51–60	4(17.4)
61 or more	0(0)
**Age of father**	
21–30	0(0)
31–40	10(41.7)
41–50	9(37.5)
51–60	4(16.7)
61 or more	1(4.2)
**Place of residence**	
Suburb or large urban center	9(34.6)
Medium-sized city	9(34.6)
Small city	1(3.8)
Rural area	7(26.9)

* This study targeted patients with severe OI.

Descriptive statistics on the experience obtaining a diagnosis, use of health services, use of social and psychological support and the expectations regarding specialized services are summarized in Table 2.

**Table 2:** Experiences while seeking care for OI.

Experience	Description	Unit
		**N (%)**
**Time to diagnosis**	OI suspected either at or within the first 3 months of birth	18(69)
**Number of doctors consulted before final diagnosis of OI**	1–2	11(42)
	3–5	12(46)
	6–10	39(11.5)
		**[Range] (mean; SD)**
**Use of health services in the past 12 months**	Number of consultations	[0–16] (4.9;3.5)
	Number of exploratory examinations	[0–9] (3.2; 2.2)
	Number of allied health services	[1–10] (5.0;2.7)
		**N (%)**
**Where admitted for treatment**	Hospitalizations (> 8 hours)	6 (24)
	Visit to an outpatient clinic	12(54.2)
	Visit to an emergency department	2(8.7)
**Use of social and psychosocial support**	Psychological support at the time of diagnosis	8(29)
	Psychological support in the past 12 months	9(36)
**Services reported as essential in a specialized center**	Occasional care related to OI	22(84.6)
	Frequent care related to OI	17(68)
	Planning several consultations or exams on same day	17(65.4)
	Coordinating information sharing between professionals	21(80.8)
	Managing transitions in patient care	18(69.2)
	Informing patients about their rights	20(76.9)
	Creating materials for others	18(69.2)
	Collaborating with research teams working on OI	20(76.9)
	Monitoring the current needs of the patient community	16(61.5)
	Training local professionals in responding to needs	20(76.9)
	Fostering dialogue and information sharing	19(73.1)
	Communicating with other specialized centers and professional networks	22(84.6)

### Experience of diagnosis

In the majority of families (70%; n = 18) OI was suspected at birth or within the first 3 months after birth. In a little more than a quarter of families (30%; *n* = 8) the reason for a misdiagnosis was a suspicion of child abuse, leading in one case to an intervention.

### Use of health services

More than 50% of families consulted in the following specialties: orthopedics, emergency services, genetics, pediatrics, nursing services and physiotherapy/rehabilitation.

In general, access to care seemed easy except for 2 families who reported that occupational therapy, dental and psychiatric services were either difficult or very difficult to access. Furthermore, whereas families were generally satisfied with their care, 2 other families reported that dental and dietician consultations poorly met their expectations because they had no referrals or the locations were too far away.

### Use of social and psychological support

Seventy percent of families did not receive psychological support at the time of diagnosis. When psychological support was provided at the time of diagnosis, it was generally provided by a medical specialist or psychologist. Almost all of the families (7 out of 8) who received psychological support thought it should be provided routinely, starting with diagnosis and throughout the life span.

### Expectations regarding specialized services and participation in research

More than 70% of families reported high expectations for specialized services to help them care for their children, plan and coordinate services from multiple specialists, manage transitions, train professionals, build awareness and information sharing on the condition.

Fifteen families (58%) had been invited to participate in research on OI in the 12 months prior to the study. Among these families, 13 were invited 2–5 times. Study procedures included, but were not limited to, participating in face-to-face interviews (*n* = 11), completing questionnaires (*n* = 9), doing physical exercises (*n* = 7 families), taking medication (*n* = 9). Almost all families (96%; *n* = 25), were supportive of research because of its potential contribution to the treatment of their child.

### Impact on family

Results from the pre-test indicate that the scales had good internal consistency as shown by the Cronbach alphas for the Impact on Family (IOF) Scale and the Family/Social (FS) and Personal Strain (PS) subscales. In addition, the means, standard deviations and ranges obtained suggest a moderately low level of impact on families with variability in the range of impacts. See Table 3.

**Table 3:** Impact on Family.

Dimension	No. of items	Alpha coefficient	Mean	S.D.	Min.	Max.
Familial (FS)	9	.723	28.7	5.12	19	36
Personal Strain^a^ (PS)	5	.821	14.7	4.18	7	20
Total score (IOF)	14	.864	43.9	8.72	28	56

^a^The Personal Strain subscale used in the study is an abbreviated version and does not include the question: «Traveling to the hospital is a strain on me”.

About half of respondents (48%) had a net family income of $50,000 or less before the diagnosis and only a small proportion (8%; *n* = 2) experienced a drop in net family income after the diagnosis. In addition, few (19%; *n* = 7) families were forced to move because of their child’s disease, and those who moved did so mainly to live in accommodation that was better suited to their needs. While fifteen families (40%) reported that the diagnosis did not affect their marital life, three families reported a negative effect.

### Feedback from families on the questionnaire

Upon reception of the completed tool, the RA successfully obtained 18 follow-up evaluation forms with questions about how long it took to complete the questionnaire and the clarity of items. Results of the follow-up evaluation are displayed in Table 4.

**Table 4:** Feedback from families on the questionnaire (n = 18)

ID	Needed assistance to complete tool^a^	Items are clear^b^	Time to complete the tool (min.)	Items are repetitive^a^	Items forgotten^a^	Tool was useful^a^	Comments
43	2	2	15	2	2	1	No comment
44	2	2	30	2	2	1	No comment
9	2	1	20	2	2	1	No comment
7	2	1	15	2	1	1	There were a lot of changes in her daughter's condition since the beginning so some questions were not pertinent
3	1	2	30	2	2	1	Needed the help of his wife to remember dates
4	2	2	20	2	2	1	The questions on income helps to realize the loss of income
35	2	2	15	2	2	1	Always happy to be part of research
46	2	2	60	2	2	1	Misdiagnosis is a big problem; hope this research will help future children.
39	2	2	60	2	2	1	No comment
49	2	1	20	2	2	1	No comment
64	2	3	60	2	2	1	Chose to fill the questionnaires in the waiting room. Needed to read questions several times.
53	1	1	20	2	2	1	Any research helps families
60	2	1	30	2	2	1	Questions about salary is personal
38	2	2	60	1	2	1	Filled with husband
58	2	2	30	2	2	1	Thank you for taking time to do this research
50	2	1	20	2	2	1	No comment
65	2	2	25	2	2	1	No comment
59	2	1	20	2	2	1	No comment

^a^1 = yes/2 = no.

^b^1 = yes/2 = somewhat/3 = no.

### Reflective thoughts on patient involvement

The tool development process involving knowledge users (clinicians, patients and caregivers), raised two main challenges. First, it points to the need for a clearly developed process for acquainting patients with research methodology to ensure their full participation in the more technical aspects of the research and development process. Second, it suggests a need, at the institutional level, for a compensatory policy for patients who contribute to research projects. Despite these challenges, the administrative support staff (of the SHC) facilitated patient and caregiver involvement in the development of the tool.

## Discussion

This study used an IKT approach to involve knowledge users (clinicians, a caregiver of and a patient living with OI) in the development of a tool to map out the experiences of patients with OI and their families, and to understand their expectations and the challenges they face in seeking diagnosis and care. Findings from the study lead to three main observations.

First, involving a patient and a caregiver in the development process enables the inclusion of their experiential knowledge with OI in the development of the tool [22]. Although their involvement was valued and consisted of revising the items, they were not involved in the initial phases of the questionnaire development, namely content mapping and item generation. We acknowledge that this level of involvement for patients and caregivers is sub-optimal and does not represent patient-led research, which is the gold standard in patient involvement in research [23, 22]. However, our experience shows that increased organizational and administrative support would be required to facilitate greater patient involvement; this includes the development of a compensatory policy and offering training sessions to develop the research skills of patients [24, 23].

Second, the tool provides a comprehensive picture of the family and the patient experience in obtaining a diagnosis and their satisfaction with medical services. Findings from the pre-test showed that although most families have a generally “good” experience (i.e. in obtaining a diagnosis and in their satisfaction with the process and the services they receive), a small number report major difficulties (i.e. investigated by authorities, consulted a large number of doctors, received many services, expectations not met). This finding is in contrast to other studies on RGDs that report long delays in obtaining a definitive diagnosis. This may be due to the fact that the first clinical symptoms of severe OI are visible physical deformities, which provide clear cues to aid in diagnosis. It may also be due to the fact that the study took place at the SHC, which is a tertiary reference center for the treatment of OI. It could also be due to the fact that a large proportion of the parents had been invited to participate in other research studies and thereby might have gained knowledge about how to navigate the health care system. Therefore, population-wide studies are needed to understand the diagnosis experience by the broader population of OI patients and their families. Finally, 8% of respondents were reported for child abuse. This rate may be higher in the general population of families with OI, particularly the families of children with mild cases. With regard to the tool itself, its psychometric properties are satisfactory: the construct validity of the questionnaire was enhanced by the use of a theoretical framework, the content validity was confirmed by revision by content experts, and the criterion validity was established through comparison to the IOF[25]. However, following the pre-test families reported that improvements to the layout, length, and item clarity could promote its routine use. The feasibility of an online version is currently under consideration.

Third, based on the expectations of people with OI, several areas can be targeted to improve the care of people living with OI. Because of the complicated and long-term nature of the disease and the psycho-social and financial burden it places on families [5], the management of OI requires a multidisciplinary approach that not only includes clinicians, but also social and support services throughout the life course [26]. Thus, collaborative health service organizations are to be encouraged. Moreover, the most efficient model of health care delivery to date seems to be specialized tertiary centers with local clinicians and support staff who are knowledgeable about the disease [27].

Finally, we argue that families should be empowered to be engaged in the management of their conditions and develop research endeavors that they value as having a significant impact on the quality of their life and health. Patient engagement in care and research should be supported in OI as well as in other RGDs. Creating opportunities for knowledge providers and knowledge users to exchange on research questions and set priorities through forums and information days would be a starting point to support IKT initiatives with patients with RGDs and their families.

## Conclusions

The IKT process used in this study included the participation of knowledge users in the development of a questionnaire that provides a unique insight into the experiences of patients and families with OI and the psycho-social impact of the illness. The use of ICF as a theoretical framework that integrates medical and contextual aspects of a person’s health condition may facilitate the application of the questionnaire in a broad range of cultural settings. Preliminary analyses demonstrated the questionnaire has sound psychometric properties. Further study is needed to test the utility of the questionnaire for the broader population of OI patients and their families, and for other RGDs.
